# *XPG* gene polymorphisms and cancer susceptibility: evidence from 47 studies

**DOI:** 10.18632/oncotarget.16146

**Published:** 2017-03-13

**Authors:** Jiawen Huang, Xiaoqi Liu, Ling-Ling Tang, Jian-Ting Long, Jinhong Zhu, Rui-Xi Hua, Jufeng Li

**Affiliations:** ^1^ Department of Pharmacy, The First Affiliated Hospital of Jinan University, Guangzhou 510630, Guangdong, China; ^2^ Department of Pharmacy, Guangdong General Hospital, Guangdong Academy of Medical Sciences, Guangzhou 510080, Guangdong, China; ^3^ School of Public Health, Sun Yat-sen University, Guangzhou 510060, Guangdong, China; ^4^ Department of Oncology, The First Affiliated Hospital of Sun Yat-sen University, Guangzhou 510080, Guangdong, China; ^5^ Molecular Epidemiology Laboratory and Department of Laboratory Medicine, Harbin Medical University Cancer Hospital, Harbin 150040, Heilongjiang, China

**Keywords:** *XPG*, polymorphism, cancer, meta-analysis

## Abstract

Xeroderma pigmentosum group G (XPG) is a single-strand-specific DNA endonuclease that functions in the nucleotide excision repair pathway. Genetic variations in *XPG* gene can alter the DNA repair capacity of this enzyme. We evaluated the associations between six single nucleotide polymorphisms (SNPs) in *XPG* (rs1047768 T>C, rs2296147 T>C, rs2227869 G>C, rs2094258 C>T, rs751402 C>T, and rs873601 G>A) and cancer risk. Forty-seven studies were identified in searches of the PubMed, Scopus, Web of Science, China National Knowledge Infrastructure, and WanFang databases. Crude odds ratios (ORs) and 95% confidence intervals (CIs) were calculated using a fixed or random effects model. We found that rs873601 G>A was associated with an increased overall cancer risk (AA vs. GG: OR = 1.14, 95% CI = 1.06–1.24; GA/AA vs. GG: OR = 1.08, 95% CI = 1.02–1.15; A vs. G: OR = 1.06, 95% CI = 1.02–1.10). In a stratified analysis, rs1047768 T>C was associated with an increased risk of lung cancer, rs2227869 G>C was associated with a decreased risk of cancer in population-based studies, and rs751402 C>T and rs873601 G>A were associated with the risk of gastric cancer. Our data indicate that rs873601 G>A is associated with cancer susceptibility.

## INTRODUCTION

There were an estimated 14.1 million new cancer cases and 8.2 million cancer-related deaths in 2012 worldwide [1, 2]. Although recent advances in the diagnosis and treatment of various cancers have improved patient prognosis, most malignancies still impose a heavy burden on society. Cancer is a multifactorial, chronic disease caused by both endogenous (genetic, immune, and endocrine disorders) and exogenous factors (environmental carcinogens and unhealthy behaviors) [1]. Among these etiological factors, gene-environment interactions have been shown to play key roles in cancer development.

The maintenance of genomic integrity is essential for human health. However, DNA damage can occur due to exposure to various chemicals, environmental agents, and ultraviolet radiation. DNA damage can also occur naturally. For example, metabolic processes can generate compounds that damage DNA, which include reactive oxygen and reactive nitrogen species. There are five major DNA damage repair pathways in humans: nucleotide excision repair (NER), base excision repair, double-strand break repair, mismatch repair, and homologous recombination [3]. Failure to properly repair DNA damage can lead to tumorigenesis. The versatile NER pathway is responsible for excising DNA lesions including cross-links, bulky adducts, thymidine dimers, alkylating damage, and oxidative DNA damage [3].

There are at least eight core functional genes in the NER pathway. These include Excision repair cross complementing group 1 (*ERCC1*) and Xeroderma pigmentosum group (*XP*) *A-G*. *XPG*, also known as *ERCC5*, is located on chromosome 13q22-q33 [4]. The *XPG* gene encodes a single-strand specific DNA endonuclease of 1,186 amino acids that cleaves the damaged DNA strand at the 3’ end [5]. Defects in the *XPG* gene can impair DNA repair resulting in genomic instability and carcinogenesis [6]. Single nucleotide polymorphisms (SNPs) in the *XPG* gene have been associated with various cancers including colorectal [7], lung [8, 9], gastric [10, 11], and laryngeal [12]. However, different studies have achieved conflicting results. For example, Duan et al. found that rs2296147 T>C in *XPG* was associated with an increased risk of gastric cancer [13], but this association was not replicated in other studies [10, 11]. The discordances might be attributed to the limited sample sizes of individual studies, different sources of controls, and ethnic variation. In this study, we performed a meta-analysis of the associations between six potentially functional SNPs: rs1047768 T>C, rs2296147 T>C, rs2227869 G>C, rs2094258 C>T, rs751402 C>T, and rs873601 G>A in the *XPG* gene and the risk of cancer.

## RESULTS

### Study characteristics

**Figure 1:**
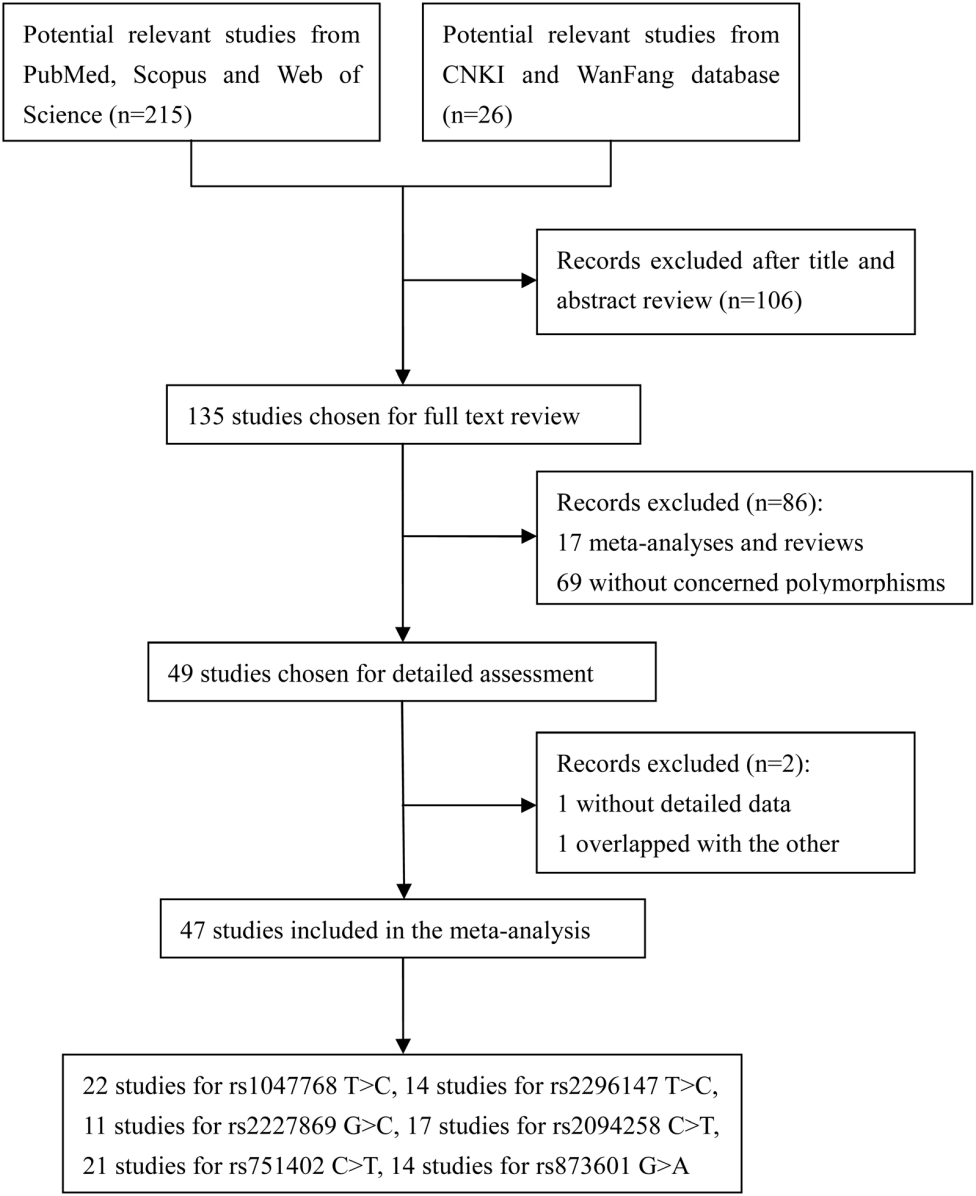
Flow diagram showing the process used to identify eligible studies

A total of 215 articles were identified using the Web of Science, Scopus, and PubMed. An additional 26 potential relevant articles were identified in the CNKI and WanFang databases. After screening the titles and abstracts, 135 studies remained for further full-text review. We excluded 17 meta-analyses and reviews as well as 69 studies that did not assess the SNPs of interest. A detailed assessment was then performed of 49 studies. Two of these studies were removed, one because there was a lack of detailed genotype data and the other because of study population overlap. The final meta-analysis included 47 articles. There were 22 articles with 12,833 cases and 151,86 controls for rs1047768 T>C [7–9, 12, 14–31], 14 studies with 11,327 cases and 12,684 controls for rs2296147 T>C [9–11, 13, 18, 24, 26–28, 32–37], 11 studies with 5,898 cases and 7,448 controls for rs2227869 G>C [8, 9, 14, 17, 18, 20, 22, 25, 38–40], 17 studies with 9,826 cases and 10,552 controls for rs2094258 C>T [10, 11, 18, 24, 26–28, 34–37, 41–46], 21 studies with 10,369 cases and 11,207 controls for rs751402 C>T [10, 13, 24, 26–29, 31, 32, 36, 37, 42–45, 47–52], and 14 studies with 10,873 cases and 12,535 controls for rs873601 G>A [9–11, 18, 24, 26–28, 32, 34, 36, 52–54]. A flow chart summarizing the process of relevant study identification is shown in Figure 1, and the study characteristics are shown in Table 1.

**Table 1:** Characteristics of the studies included in the meta-analysis

Author	Year	Country	Ethnicity	Source	Cancer	Case	Control							MAF	HWE	Score
						BB	Bb	bb	All	BB	Bb	bb	All			
**rs1047768 T>C**																
Shen M	2005	China	Asian	PB	Lung	55	49	14	118	63	36	13	112	0.28	0.037	10
Zienolddiny S	2006	Norway	Caucasian	PB	Lung	60	119	137	316	109	126	138	373	0.54	<0.001	11
Moreno V	2006	Spain	Caucasian	HB	Colorectal	114	184	53	351	105	164	51	320	0.42	0.325	11
Garcia-Closas M	2006	Spain	Caucasian	HB	Bladder	188	530	385	1103	222	506	366	1094	0.57	0.052	12
Xie WM	2007	China	Asian	PB	HCC	194	195	38	427	235	196	48	479	0.30	0.451	11
Abbasi R	2009	Germany	Caucasian	PB	Laryngeal	43	127	78	248	115	320	212	647	0.57	0.762	13
Hussain SK	2009	China	Asian	PB	Gastric	97	61	12	170	189	168	29	386	0.29	0.173	13
Ma H	2012	USA	Caucasian	HB	SCCHN	184	506	369	1059	179	507	379	1065	0.59	0.669	11
Sakoda LC	2012	USA	Caucasian	PB	Lung	108	378	256	742	245	722	507	1474	0.59	0.656	15
He J	2013	China	Asian	HB	Gastric	571	469	85	1125	610	474	112	1196	0.29	0.155	13
Paszkowska-Szczur K	2013	Poland	Caucasian	PB	Melanoma	128	291	214	633	242	623	465	1330	0.58	0.189	13
Li X	2014	China	Asian	HB	Laryngeal	49	101	60	210	46	97	67	210	0.55	0.333	9
Mirecka A	2014	Poland	Caucasian	HB	Prostate	128	272	221	621	154	368	259	781	0.57	0.260	9
Li XC	2014	China	Asian	HB	Gastric	37	95	85	217	29	93	95	217	0.65	0.414	8
Na N	2015	China	Asian	HB	Breast	161	140	24	325	171	134	20	325	0.27	0.352	10
Paszkowska-Szczur K	2015	Poland	Caucasian	HB	Colorectal	104	221	138	463	242	623	465	1330	0.58	0.189	9
He J	2016	China	Asian	HB	Neuroblastoma	135	93	20	248	307	198	26	531	0.24	0.409	10
Hua RX	2016	China	Asian	HB	Colorectal	970	758	173	1901	1023	812	142	1977	0.28	0.266	10
Hua RX	2016	China	Asian	HB	Gastric	607	445	90	1142	625	461	87	1173	0.27	0.875	11
Li RJ	2016	China	Asian	HB	Gastric	57	92	67	216	68	87	61	216	0.48	0.004	7
Wang MY	2016	China	Asian	HB	Prostate	491	433	80	1004	534	440	81	1055	0.29	0.461	10
Bai Y	2016	China	Asian	HB	Gastric	41	98	55	194	32	106	87	225	0.62	0.975	6
**rs2296147 T>C**																
Shao MH	2007	China	Asian	HB	Lung	570	304	52	926	590	358	31	979	0.21	0.008	10
Doherty JA	2011	USA	Mixed	PB	Endometrial	194	356	165	715	199	364	157	720	0.47	0.696	11
Duan Z	2012	China	Asian	HB	Gastric	257	122	24	403	260	132	11	403	0.19	0.232	11
He J	2012	China	Asian	HB	Gastric	700	371	54	1125	742	398	56	1196	0.21	0.779	13
Ma H	2012	USA	Caucasian	HB	SCCHN	280	532	244	1056	294	543	228	1065	0.47	0.440	11
Sakoda LC	2012	USA	Caucasian	PB	Lung	182	385	174	741	407	723	341	1471	0.48	0.565	15
Zhu ML	2012	China	Asian	HB	ESCC	757	305	53	1115	699	368	50	1117	0.21	0.860	13
Yang WG	2012	China	Asian	HB	Gastric	208	105	24	337	196	110	41	347	0.28	<0.001	9
Yang B	2013	China	Asian	HB	Prostate	37	49	143	229	25	46	167	238	0.80	<0.001	8
Na N	2015	China	Asian	HB	Breast	188	104	33	325	199	98	28	325	0.24	0.003	9
Sun Z	2015	China	Asian	HB	NPC	119	177	76	372	111	180	80	371	0.46	0.660	11
Chen YZ	2016	China	Asian	HB	Gastric	442	217	33	692	475	264	32	771	0.21	0.535	11
He J	2016	China	Asian	HB	Neuroblastoma	160	79	9	248	343	170	18	531	0.19	0.583	10
Hua RX	2016	China	Asian	HB	Colorectal	1169	644	88	1901	1213	692	72	1977	0.21	0.027	9
Hua RX	2016	China	Asian	HB	Gastric	725	364	53	1142	746	388	39	1173	0.20	0.182	11
**rs2227869 G>C**																
Shen M	2005	China	Asian	PB	Lung	103	14	1	118	100	11	0	111	0.05	0.583	11
Garcia-Closas M	2006	Spain	Caucasian	HB	Bladder	1050	91	2	1143	1046	90	0	1136	0.04	0.164	12
Huang WY	2006	USA	Caucasian	PB	Colorectal	598	52	1	651	601	60	1	662	0.05	0.694	14
Hooker S	2008	USA	African	HB	Prostate	234	20	0	254	274	27	0	301	0.05	0.415	7
Hussain SK	2009	China	Asian	PB	Gastric	174	13	0	187	314	56	3	372	0.08	0.773	13
Ma H	2012	USA	Caucasian	HB	SCCHN	987	70	2	1059	974	90	2	1066	0.04	0.958	11
Sakoda LC	2012	USA	Caucasian	PB	Lung	1	63	680	744	2	110	1362	1474	0.96	0.886	15
Santos LS	2013	Portugal	Caucasian	HB	Thyroid	99	6	1	106	184	27	1	212	0.02	0.993	8
Paszkowska-Szczur K	2013	Poland	Caucasian	PB	Melanoma	567	67	2	636	1168	162	2	1332	0.06	0.137	13
Mirecka A	2014	Poland	Caucasian	HB	Prostate	485	83	3	571	682	99	1	782	0.06	0.181	9
Paszkowska-Szczur K	2015	Poland	Caucasian	HB	Colorectal	372	55	2	429	1168	162	2	1332	0.06	0.137	9
**rs2094258 C>T**																
He J	2012	China	Asian	HB	Gastric	457	518	150	1125	457	560	179	1196	0.62	0.728	13
Ma H	2012	USA	Caucasian	HB	SCCHN	706	295	37	1038	721	291	41	1053	0.82	0.092	11
Yang WG	2012	China	Asian	HB	Gastric	131	149	57	337	145	166	36	347	0.66	0.252	10
Zhu ML	2012	China	Asian	HB	ESCC	414	524	177	1115	424	525	168	1117	0.61	0.793	13
Yang B	2013	China	Asian	HB	Prostate	61	75	93	229	58	75	105	238	0.40	<0.001	9
Na N	2015	China	Asian	HB	Breast	102	157	66	325	131	147	47	325	0.63	0.581	10
Sun Y	2015	China	Asian	HB	Laryngeal	140	106	25	271	152	101	18	271	0.75	0.826	11
Sun Z	2015	China	Asian	HB	NPC	209	68	95	372	211	66	94	371	0.66	<0.001	10
Chen YZ	2016	China	Asian	HB	Gastric	287	304	101	692	291	368	112	771	0.62	0.803	11
He J	2016	China	Asian	HB	Neuroblastoma	116	93	39	248	203	254	74	531	0.62	0.701	10
Hua RX	2016	China	Asian	HB	Colorectal	797	856	248	1901	899	881	197	1977	0.68	0.378	10
Feng YB	2016	China	Asian	HB	Gastric	15	75	87	177	15	96	127	238	0.26	0.577	6
Hua RX	2016	China	Asian	HB	Gastric	499	508	135	1142	527	524	122	1173	0.67	0.623	11
Lu JJ	2016	China	Asian	HB	Gastric	17	67	100	184	13	72	121	206	0.24	0.605	6
Ma SH	2016	China	Asian	HB	Breast	27	136	157	320	15	96	127	238	0.26	0.577	7
Yang LQ	2016	China	Asian	HB	Gastric	71	74	10	155	121	111	14	246	0.72	0.076	6
Ying MF	2016	China	Asian	HB	Pancreatic	87	92	16	195	117	115	22	254	0.69	0.400	7
**rs751402 C>T**																
Shao MH	2007	China	Asian	HB	Lung	105	429	433	967	110	425	448	983	0.67	0.544	11
Yoon AJ	2011	Taiwan	Asian	HB	HCC	11	52	33	96	32	137	167	336	0.70	0.614	6
Duan Z	2012	China	Asian	HB	Gastric	47	181	172	400	29	165	206	400	0.72	0.605	11
He J	2012	China	Asian	HB	Gastric	148	491	486	1125	137	499	560	1196	0.68	0.110	13
Zavras AI	2012	Taiwan	Mixed	HB	OSCC	31	110	98	239	32	137	167	336	0.70	0.614	9
Meng X	2013	China	Asian	HB	Salivary gland	11	63	59	133	23	55	64	142	0.64	0.065	8
Na N	2015	China	Asian	HB	Breast	45	152	128	325	41	147	137	325	0.65	0.872	10
Sun Z	2015	China	Asian	HB	NPC	237	118	17	372	235	117	19	371	0.21	0.377	11
Wang H	2016	China	Asian	HB	Breast	1	10	90	101	11	39	51	101	0.70	0.398	9
Chen YZ	2016	China	Asian	HB	Gastric	93	313	286	692	89	331	351	771	0.67	0.416	11
He J	2016	China	Asian	HB	Neuroblastoma	38	114	96	248	82	241	208	531	0.62	0.380	10
Hua RX	2016	China	Asian	HB	Colorectal	248	860	792	1900	301	952	724	1977	0.61	0.680	10
Guo BW	2016	China	Asian	HB	Gastric	22	73	47	142	21	136	117	274	0.68	0.029	5
Feng YB	2016	China	Asian	HB	Gastric	24	83	70	177	28	107	101	236	0.65	0.967	6
Hua RX	2016	China	Asian	HB	Gastric	161	555	426	1142	189	551	433	1173	0.60	0.537	11
Li RJ	2016	China	Asian	HB	Gastric	22	106	88	216	18	103	95	216	0.68	0.174	8
Lu JJ	2016	China	Asian	HB	Gastric	24	91	69	184	22	97	87	206	0.66	0.510	6
Ma SH	2016	China	Asian	HB	Breast	43	150	127	320	28	101	107	236	0.67	0.580	7
Yang LQ	2016	China	Asian	HB	Gastric	33	73	49	155	32	111	103	246	0.64	0.807	6
Wang MY	2016	China	Asian	HB	Prostate	104	458	442	1004	111	467	477	1055	0.67	0.834	10
Zhou RM	2016	China	Asian	HB	Gastric	61	196	174	431	46	193	193	432	0.67	0.827	12
**rs873601 G>A**																
Shao MH	2007	China	Asian	HB	Lung	260	493	220	973	277	494	217	988	0.47	0.907	11
He J	2012	China	Asian	HB	Gastric	274	560	291	1125	327	605	264	1196	0.47	0.616	13
Ma H	2012	USA	Caucasian	HB	SCCHN	66	427	565	1058	83	411	572	1066	0.73	0.445	11
Sakoda LC	2012	USA	Caucasian	PB	Lung	51	299	392	742	107	584	783	1474	0.73	0.894	15
Yang WG	2012	China	Asian	HB	Gastric	96	163	78	337	91	164	91	346	0.50	0.333	10
Zhu ML	2012	China	Asian	HB	ESCC	314	566	235	1115	311	565	241	1117	0.47	0.601	13
Na N	2015	China	Asian	HB	Breast	99	156	70	325	109	150	66	325	0.43	0.276	10
Zhao F	2015	China	Asian	HB	Pancreatic	105	111	30	246	118	107	21	246	0.30	0.637	8
Chen YZ	2016	China	Asian	HB	Gastric	172	333	187	692	205	396	170	771	0.48	0.415	11
He J	2016	China	Asian	HB	Neuroblastoma	70	112	66	248	137	270	124	531	0.49	0.686	10
Wang B	2016	China	Asian	HB	HCC	163	271	104	538	271	408	214	893	0.47	0.014	12
Hua RX	2016	China	Asian	HB	Colorectal	476	954	471	1901	550	1025	402	1977	0.46	0.057	10
Hua RX	2016	China	Asian	HB	Gastric	311	557	274	1142	323	598	252	1173	0.47	0.424	11
Zhou RM	2016	China	Asian	HB	Gastric	115	215	101	431	132	200	100	432	0.46	0.152	12

Abbreviations: HB, hospital-based; PB, population-based; PCR-RFLP, polymerase chain reaction-restriction fragment length polymorphism; MAF, minor allele frequency; HWE, Hardy-Weinberg equilibrium; HCC, hepatocellular carcinoma; SCCHN, squamous cell carcinoma of the head and neck; ESCC, esophageal squamous cell carcinoma; OSCC, oral squamous cell carcinoma; NPC, nasopharyngeal carcinoma.

### Meta-analysis results

We observed no significant association between rs1047768 T>Cand overall cancer risk (Table 2). However, in stratified analysis, rs1047768 T>C was associated with an increased risk of lung cancer under homozygous [odds ratio (OR) = 1.32, 95% confidence interval (CI) = 1.06–1.64], heterozygous (OR = 1.35, 95% CI = 1.10–1.65), dominant (OR = 1.35, 95% CI = 1.12–1.63), and allele contrast (OR = 1.14, 95% CI = 1.02–1.27) models.

**Table 2:** Associations between the six SNPs in the *XPG* gene and cancer risk

Variables	No. of studies	No. of cases	No. of controls	Homozygous		Heterozygous		Recessive		Dominant		Allele	
				OR(95% CI)	*P* ^het^	OR(95% CI)	*P* ^het^	OR(95% CI)	*P* ^het^	OR(95% CI)	*P* ^het^	OR(95% CI)	*P* ^het^
**rs1047768 T>C**				CC vs. TT		CT vs. TT		CC vs. CT/TT		CC/CT vs. TT		C vs. T	
All	22	12833	15186	1.03 (0.95–1.11)	0.010	1.03 (0.97–1.09)	0.192	1.00 (0.93–1.07)	0.171	1.03 (0.98–1.09)	0.038	1.01 (0.98–1.05)	0.012
*Ethnicity*													
Caucasian	9	5536	7084	1.03 (0.88–1.21)	0.012	1.04 (0.95–1.14)	0.061	1.00 (0.93–1.07)	0.344	1.04 (0.90–1.20)	0.011	1.01 (0.94–1.10)	0.011
Asian	13	7297	8102	1.03 (0.92–1.16)	0.081	1.02 (0.96–1.10)	0.493	1.00 (0.90–1.11)	0.116	1.03 (0.96–1.10)	0.304	1.02 (0.97–1.07)	0.105
*Cancer type*													
Lung	3	1176	1959	**1.32 (1.06–1.64)**	0.175	**1.35 (1.10–1.65)**	0.278	1.08 (0.92–1.26)	0.360	**1.35 (1.12–1.63)**	0.172	**1.14 (1.02–1.27)**	0.059
Colorectal	3	2715	3627	0.95 (0.63–1.45)	0.006	0.96 (0.86–1.08)	0.480	0.99 (0.70–1.39)	0.012	0.94 (0.78–1.14)	0.133	0.99 (0.91–1.07)	0.020
Gastric	6	3064	3413	0.88 (0.74–1.05)	0.118	0.98 (0.88–1.09)	0.263	0.88 (0.74–1.05)	0.279	0.97 (0.87–1.07)	0.127	0.93 (0.82–1.04)	0.073
Others	10	5878	7517	1.04 (0.93–1.15)	0.507	1.05 (0.96–1.14)	0.670	1.01 (0.93–1.10)	0.725	1.05 (0.97–1.14)	0.628	1.03 (0.98–1.08)	0.659
**rs2296147 T>C**				CC vs. TT		CT vs. TT		CC vs. CT/TT		CC/CT vs. TT		C vs. T	
All	15	11327	12684	1.10 (1.00–1.12)	0.068	0.95 (0.90–1.01)	0.480	1.08 (0.99–1.18)	0.057	0.97 (0.92–1.03)	0.297	1.00 (0.96–1.04)	0.118
Gastric	5	3699	3890	1.11 (0.76–1.60)	0.026	0.95 (0.86–1.04)	0.945	1.13 (0.78–1.63)	0.025	0.96 (0.88–1.06)	0.697	0.99 (0.91–1.07)	0.197
**rs2227869 G>C**				CC vs. GG		GC vs. GG		CC vs. GC/GG		GC/CC vs. GG		C vs. G	
All	11	5898	7448	1.67 (0.82–3.41)	0.924	0.90 (0.80–1.02)	0.153	0.98 (0.73–1.32)	0.699	0.92 (0.81–1.03)	0.108	0.93 (0.83–1.04)	0.079
PB	5	2336	3951	1.08 (0.37–3.10)	0.793	**0.80 (0.65–0.99)**	0.239	0.89 (0.65–1.21)	0.766	0.81 (0.66–1.00)	0.170	**0.84 (0.71–0.99)**	0.115
HB	6	3562	4829	2.46 (0.91–6.67)	0.852	0.96 (0.82–1.11)	0.198	2.48 (0.91–6.74)	0.865	0.98 (0.84–1.13)	0.190	1.00 (0.87–1.15)	0.202
**rs2094258 C>T**				TT vs. CC		CT vs. CC		TT vs. CT/CC		CT/TT vs. CC		T vs. C	
All	17	9826	10552	1.09 (1.00–1.19)	0.025	1.00 (0.94–1.07)	0.314	1.07 (0.99–1.16)	0.089	1.02 (0.97–1.09)	0.081	1.03 (0.99–1.08)	0.015
Gastric	7	3812	4177	0.99 (0.86–1.15)	0.083	0.95 (0.86–1.05)	0.734	1.01 (0.89–1.14)	0.119	0.96 (0.88–1.06)	0.409	0.98 (0.92–1.05)	0.133
**rs751402 C>T**				TT vs. CC		CT vs. CC		TT vs. CT/CC		CT/TT vs. CC		T vs. C	
All	21	10369	11207	1.18 (1.00–1.39)	<0.001	1.10 (0.99–1.23)	0.082	1.02 (0.94–1.10)	0.006	1.11 (0.98–1.25)	<0.001	1.08 (0.98–1.18)	<0.001
Gastric	10	4664	5150	**1.38 (1.12–1.70)**	0.020	**1.14 (1.05–1.24)**	0.936	**1.27 (1.06–1.51)**	0.053	**1.17 (1.08–1.26)**	0.437	**1.17(1.07–1.27)**	0.043
**rs873601 G>A**				AA vs. GG		GA vs. GG		AA vs. GA/GG		GA/AA vs. GG		A vs. G	
All	14	10873	12535	**1.14 (1.06–1.24)**	0.193	1.06 (0.99–1.13)	0.904	1.08 (0.99–1.17)	0.035	**1.08 (1.02–1.15)**	0.841	**1.06 (1.02–1.10)**	0.234
Gastric	5	3727	3918	**1.18 (1.04–1.34)**	0.333	1.04 (0.93–1.16)	0.663	**1.16 (1.04–1.28)**	0.263	1.08 (0.98–1.20)	0.578	**1.09 (1.02–1.16)**	0.336

No significant association was observed between rs2296147 T>C and overall cancer risk. Similarly, there was no significant association between rs2227869 G>C and overall cancer risk. However, a significant association was identified in population-based studies when the data were stratified based on the source of the controls under heterozygous (OR = 0.80, 95% CI = 0.65–0.99) and allele contrast (OR = 0.84, 95% CI = 0.71–0.99) models. We observed an association between rs2094258 C>T and overall cancer risk under the homozygous model (OR = 1.09, 95% CI = 1.00–1.19), which approached borderline statistical significance. Another borderline significant association was observed between rs751402 C>T and overall cancer risk under the homozygous model (OR = 1.18, 95% CI = 1.00–1.39). In the stratified analysis, a significant association was observed for gastric cancer under homozygous (OR = 1.38, 95% CI = 1.12–1.70), heterozygous (OR = 1.14, 95% CI = 1.05–1.24), recessive (OR = 1.27, 95% CI = 1.06–1.51), dominant (OR = 1.17, 95% CI = 1.08–1.26), and allele contrast (OR = 1.17, 95% CI = 1.07–1.27) models.

**Figure 2:**
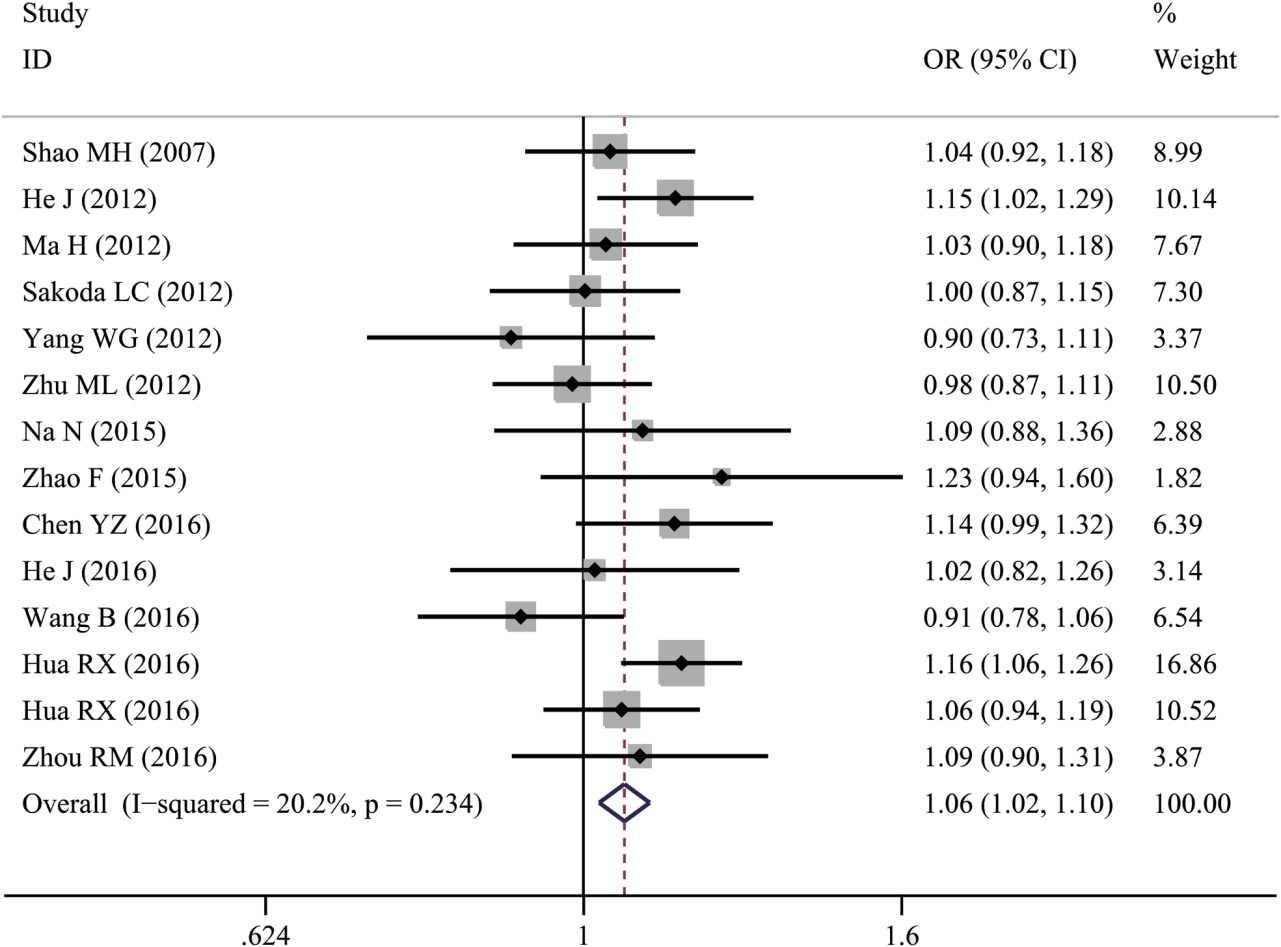
Forest plot of overall cancer risk associated with rs873601 G>A in the *XPG* gene under an allele contrast model For each study, estimated ORs and 95% CIs are plotted with a box and horizontal line, respectively. (◇, pooled ORs and associated 95% CIs).

A significant association was observed between rs873601 G>A and overall cancer risk under homozygous (OR = 1.14, 95% CI = 1.06–1.24), dominant (OR = 1.08, 95% CI = 1.02–1.15), and allele contrast (OR = 1.06, 95% CI = 1.02-1.10) models (Figure 2). The association with gastric cancer remained statistically significant under homozygous (OR = 1.18, 95% CI = 1.04–1.34), recessive (OR = 1.16, 95% CI = 1.04–1.28), and allele contrast (OR = 1.09, 95% CI = 1.02–1.16) models.

### Heterogeneity and sensitivity analysis

Study heterogeneity was observed for the association between rs1047768 T>C and overall cancer risk under homozygous, dominant, and allele contrast models (*P* = 0.010, *P* = 0.038, and *P* = 0.012, respectively); rs2094258 C>T under homozygous and allele contrast models (*P* = 0.025 and *P* = 0.015, respectively); rs751402 C>T under homozygous, recessive, dominant, and allele contrast models (*P* < 0.001, *P* = 0.006, *P* < 0.001, *P* < 0.001, respectively); and rs873601 G>A under a recessive model (*P* = 0.035). These data indicated that the removal of any individual study from the analysis did not qualitatively change the pooled ORs (data not shown).

### Publication bias

**Figure 3:**
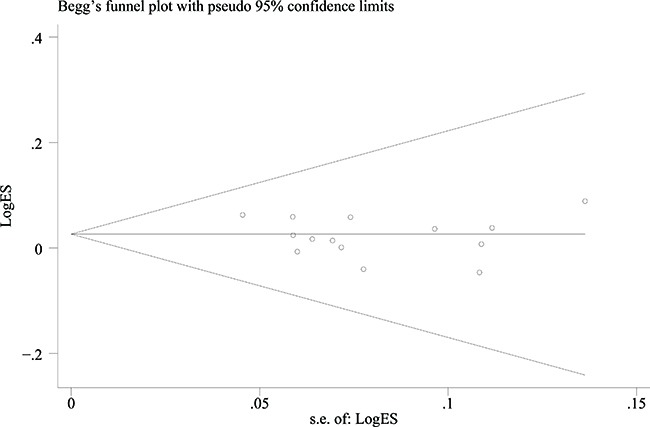
Funnel plot of the association between rs873601 G>A in the *XPG* gene and overall cancer risk under an allele contrast model Each point represents an individual study that reported the indicated association.

The Begg's funnel plots of the associations between the SNPs in the *XPG* gene and cancer risk were basically symmetrical (Figure 3). Egger's tests indicated there was no publication bias for rs1047768 T>C under homozygous (*P* = 0.107), heterozygous (*P* = 0.190), recessive (*P* = 0.325), dominant (*P* = 0.137), and allele contrast (*P* = 0.301) models; rs2296147 T>C under homozygous (*P* = 0.789), heterozygous (*P* = 0.925), recessive (*P* = 0.577), dominant (*P* = 0.464), and allele contrast (*P* = 0.129) models; rs2227869 G>C under homozygous (*P* = 0.708), heterozygous (*P* = 0.289), recessive (*P* = 0.042), dominant (*P* = 0.297), and allele contrast (*P* = 0.197) models; rs2094258 C>T under homozygous (*P* = 0.387), heterozygous (*P* = 0.350), recessive (*P* = 0.844), dominant (*P* = 0.276), and allele contrast (*P* = 0.351) models; rs751402 C>T under homozygous (*P* = 0.107), heterozygous (*P* = 0.336), recessive (*P* = 0.137), dominant (*P* = 0.325), and allele contrast (*P* = 0.301) models; and rs873601 G>A under homozygous (*P* = 0.395), heterozygous (*P* = 0.656), recessive (*P* = 0.645), dominant (*P* = 0.811), and allele contrast (*P* = 0.346) models (Table 3).

**Table 3:** Publication bias among studies that evaluated the associations between the six SNPs in the *XPG* gene and cancer susceptibility

Polymorphism	No. of studies	Egger's test *P* values				
		Homozygous	Heterozygous	Recessive	Dominant	Allele contrast
rs1047768	22	0.107	0.190	0.325	0.137	0.301
rs2296147	15	0.789	0.925	0.577	0.464	0.129
rs2227869	11	0.708	0.289	0.042	0.297	0.197
rs2094258	17	0.387	0.350	0.844	0.276	0.351
rs751402	21	0.107	0.336	0.137	0.325	0.301
rs873601	14	0.395	0.656	0.645	0.811	0.346

### False-positive *report probability (FPRP) analysis and* trial sequential analysis (TSA)

**Figure 4:**
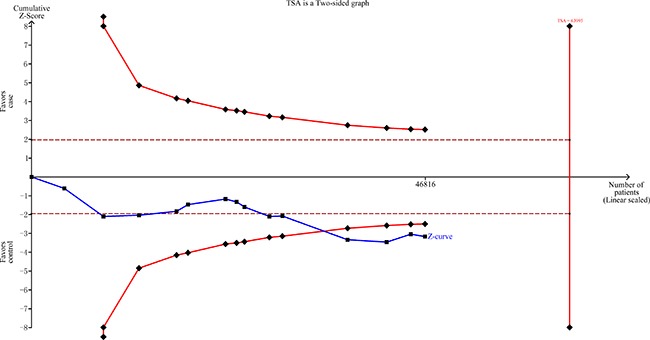
TSA of rs873601 G>A in the *XPG* gene and overall cancer risk under an allele contrast model

All significant findings remained significant at a prior probability of 0.1, with all the FPRP values less than 0.20 with the exception of the population-designed studies of rs2227869 G>C (Table 4). TSA indicated that the cumulative z-curve crossed the trial sequential monitoring boundary, suggesting that the sample size was sufficient and that no further analysis was required to confirm the results (Figure 4).

**Table 4:** False-positive report probability values for significant results

Genotype	Crude OR (95% CI)	*P* ^a^	Statistical power ^b^	Prior probability				
				0.25	0.1	0.01	0.001	0.0001
rs1047768 T>C (lung cancer)								
CC vs. TT	1.32 (1.06–1.64)	0.012	0.998	**0.035**	**0.097**	0.542	0.923	0.992
CT vs. TT	1.35 (1.10–1.65)	0.004	0.995	**0.011**	**0.033**	0.273	0.791	0.974
CC/CT vs. TT	1.35 (1.12–1.63)	0.002	0.859	**0.006**	**0.019**	**0.177**	0.685	0.956
C vs. T	1.14 (1.02–1.27)	0.017	1.000	**0.048**	**0.130**	0.622	0.943	0.994
rs2227869 G>C (population-based studies)								
GC vs. GG	0.80 (0.65–0.99)	0.041	0.987	**0.111**	0.272	0.805	0.976	0.998
C vs. G	0.84 (0.71–0.99)	0.041	1.000	**0.110**	0.271	0.803	0.976	0.998
rs751402 C>T (gastric cancer)								
TT vs. CC	1.38 (1.12–1.70)	0.002	1.000	**0.007**	**0.019**	**0.179**	0.687	0.956
CT vs. CC	1.14 (1.05–1.24)	0.003	1.000	**0.008**	**0.024**	0.213	0.732	0.965
TT vs. CT/CC	1.27 (1.06–1.51)	0.010	1.000	**0.030**	**0.085**	0.506	0.912	0.990
CT/TT vs. CC	1.17 (1.08–1.26)	<0.001	1.000	**0.001**	**0.002**	**0.019**	**0.161**	0.658
T vs. C	1.17 (1.07–1.27)	0.001	1.000	**0.002**	**0.006**	**0.063**	0.404	0.871
rs873601 G>A (overall)								
AA vs. GG	1.14 (1.06–1.24)	0.001	1.000	**0.002**	**0.006**	**0.061**	0.394	0.867
GA/AA vs. GG	1.08 (1.02–1.15)	0.012	1.000	**0.036**	**0.101**	0.552	0.926	0.992
A vs. G	1.06 (1.02–1.10)	0.002	1.000	**0.006**	**0.016**	**0.155**	0.650	0.949
rs873601 G>A (gastric cancer)								
AA vs. GG	1.18 (1.04–1.34)	0.009	1.000	**0.027**	**0.078**	0.482	0.904	0.989
AA vs. GA/GG	1.16 (1.04–1.28)	0.008	1.000	**0.022**	**0.064**	0.431	0.884	0.987
A vs. G	1.09 (1.02–1.16)	0.011	1.000	**0.031**	**0.089**	0.517	0.915	0.991

^a^Chi-square tests were used to assess the genotype frequency distributions.

^b^Statistical power was calculated using the number of observations in the subgroup and the *P* values in this table.

## DISCUSSION

The NER pathway is critical for the repair of bulky DNA lesions resulting from exposure to chemical carcinogens as well as ionizing radiation in order to maintain genomic integrity and prevent carcinogenesis [55]. Because the *XPG* gene is an indispensable component of the NER pathway, SNPs in *XPG* may alter the expression or function of XPG thereby modifying the risk of cancer. Most previous meta-analyses of the association between SNPs in *XPG* and cancer risk have focused on rs17655 G>C [56–59]. However, recent studies have shown that other SNPs in *XPG* may also be associated with cancer risk. For example, Chen et al. found that rs873601 G>A was associated with an increased risk of gastric cancer in a Chinese Han population [36]. Wang et al. found that rs751402 C>T was protective against breast cancer in Chinese Han women [47]. Additionally, the T allele of rs2296147 was associated with an increased risk of prostate cancer [35]. However, the results of previous studies have been inconsistent, possibly due to variations in the study populations and limited sample sizes. We therefore performed a meta-analysis of 47 studies to comprehensively evaluate the associations between six SNPs in *XPG*: rs1047768 T>C, rs2296147 T>C, rs2227869 G>C, rs2094258 C>T, rs751402 C>T, and rs873601 G>A and cancer risk.

The rs873601 G>A polymorphism is located in a miRNA binding site in the *XPG* gene. Thus, it may alter *XPG* expression by modulating the miRNA-mRNA interaction, which could play a role in carcinogenesis [10]. We demonstrated that rs873601 G>A was significantly associated with overall cancer risk. Individuals with the AA genotype of rs873601 had a 1.14-fold higher risk of cancer compared to individuals with the GG genotype. Similar results were obtained for gastric cancer. The A allele of rs873601 was previously shown to result in reduced mRNA expression of *XPG* in both adjacent normal gastric cancer tissue and normal cell lines in a recessive manner [10]. These findings provide insight into the molecular mechanisms by which the AA genotype of rs873601 may increase the risk of gastric cancer.

The rs751402 C>T polymorphism is located in the E2F1/YY1 binding and response site in the proximal promoter region of *XPG* [60]. This variant might reduce the DNA repair capacity of XPG by disrupting the DNA binding motifs and altering transcription factor affinities [47]. In our study, rs751402 C>T was significantly associated with overall cancer risk. The TT genotype of rs751402 was associated with an 18% increase in cancer risk compared to the CC genotype. Moreover, a significant association was observed between rs751402 C>T and gastric cancer risk under all genetic models. The rs751402 C>T polymorphism is likely to influence cancer risk by regulating *XPG* expression, but its effect on XPG function is not yet clear [47].

The rs2094258 C>T polymorphism is located in a transcription factor binding site in the 5’ region of the *XPG* gene. We found that the association between rs2094258 C>T and overall cancer risk was borderline significant. Individuals with the TT genotype of rs2094258 had a 9% higher risk of cancer compared to those with the CC genotype. However, the association was not significant in gastric cancer, indicating that it may not impact gastric cancer risk. Significant associations were observed among some subgroups for all other selected SNPs. We found that the C allele of rs1047768 may increase the risk of lung cancer. Moreover, the C allele of rs2227869 significantly reduced cancer risk in population-based studies. No statistically significant association was observed between rs2296147 T>C and overall cancer risk.

Although we found significant associations between SNPs in the *XPG* gene and cancer risk, our study had several limitations. First, although Egger's tests showed no obvious publication bias, some bias was unavoidable since only studies published in English and Chinese were included in our meta-analysis. Second, we observed significant heterogeneity in some of our analyses, which is a common drawback of a meta-analysis. Third, due to a lack of sufficient individual data, we were unable to perform multivariate analysis with adjustment for potential confounding factors such as tobacco use, alcohol consumption, and other carcinogenic factors.

Our study is the first meta-analysis of the association between the six selected SNPs in *XPG* gene and cancer risk. The results indicate that the AA genotype of rs873601 increases overall cancer risk. Additionally, rs751402 C>T and rs873601 G>A were associated with gastric cancer risk. Finally, rs1047768 T>C was found to confer susceptibility to lung cancer. Further epidemiological investigations with larger sample sizes are warranted to validate our findings. Functional studies are also required to elucidate the mechanisms by which these SNPs modify cancer risk.

## MATERIALS AND METHODS

### Study identification

We searched multiple databases including PubMed, Scopus, Web of Science, CNKI, and the WanFang database using combinations of keywords such as “*XPG*”, “polymorphism”, and “cancer” as well as synonyms “Xeroderma pigmentosum group G, ERCC5 or Excision repair cross complementing group 5”, “variant or variation”, and “tumor, neoplasm, or carcinoma”. Human studies published before December 20, 2016 in either English or Chinese were included. The reference lists in eligible studies and review articles were examined in order to identify additional relevant studies. In cases of study population overlap, the study with the largest sample size was selected.

### Inclusion and exclusion criteria

All studies included in this analysis were required to meet the following criteria: (1) study of the associations between any of the six potentially functional SNPs: rs1047768 T>C, rs2296147 T>C, rs2227869 G>C, rs2094258 C>T, rs751402 C>T, and rs873601 G>A in the *XPG* gene and cancer risk; (2) case-control study; and (3) sufficient genotype data available to calculate ORs and 95% CIs. The exclusion criteria were: (1) studies conducted in the same or overlapping population and (2) review article or conference report.

### Data extraction

Key information was independently extracted from eligible studies by two investigators and included the following items: the first author, year of publication, type of cancer, country, ethnicity, control source, number of cases and controls, the quantity of each genotype in cases and controls, minor allele frequency (MAF), and the Hardy-Weinberg equilibrium (HWE) test *P* value for the control subjects. Disagreements regarding these items were resolved through discussion.

### Statistical analysis

Chi-square tests were used to test deviation from HWE in the study control groups. Genetic associations between the six selected SNPs in the *XPG* gene and cancer risk were assessed using the crude ORs and corresponding 95% CIs under homozygous, heterozygous, recessive, dominant, and allele contrast models. Heterogeneity between studies was assessed using the Q and *I*^2^ values. A random effects model was adopted to calculate the pooled OR and 95% CI in the case of *P*^het^ < 0.1 or I^2^ > 50%. Otherwise, a fixed effects model was applied. Stratified analyses were conducted by ethnicity (Asians and Caucasians), source of control [population-based (PB) or hospital-based (HB)], and cancer type.

Sensitivity analyses were performed to assess the influence of the individual studies on the pooled OR by sequentially removing one study at a time and recalculating the pooled OR. Egger's tests were used to evaluate publication bias. FPRP analysis [61, 62] and TSA were performed as described previously [63]. All statistical analyses were performed using the STATA 12.0 software (Stata Corporation, College Station, TX, USA). All statistics were two-sided. *P* values < 0.05 were considered statistically significant.
